# Risk of osteoporosis in patients with erectile dysfunction: A PRISMA-compliant systematic review and meta-analysis

**DOI:** 10.1097/MD.0000000000026326

**Published:** 2021-06-18

**Authors:** Jiangnan Xu, Chao Wang, Yuhui Zhang, Zekun Xu, Jun Ouyang, Jianglei Zhang

**Affiliations:** a Department of Urology, The First Affiliated Hospital of Soochow University; b Soochow University, Suzhou, China.

**Keywords:** erectile dysfunction, osteoporosis, systematic review and meta-analysis

## Abstract

**Background:**

Erectile dysfunction (ED) and osteoporosis are both common health problems and have similar risk factors. Recent studies have found that people with ED have a higher risk of osteoporosis.

We aimed to systematically assess osteoporosis risk in patients with ED.

**Methods:**

A systematically research was carried out in Medline via PubMed, Cochrane Library, EMBASE, and Web of Science up to June 4, 2020, to identify articles related to ED and osteoporosis. The 2 researchers independently reviewed the literature, extracted the data, and evaluated the quality of the literature. All analyses were done using RevMan5.3 and Stata14.

**Results:**

A total of 4 studies involving 22,312 participants were included. The meta-analysis results showed that the risk of osteoporosis in the ED group was significantly higher than that in the non-ED group [odds ratio (OR) = 2.66, 95% confidence interval (95% CI) 1.42 to 4.98, *P* = .002, *I*^2^ = 68%]. Interestingly, compared with older participants, the increased risk of osteoporosis in ED patients seemed to be more pronounced in younger participants. Despite the lack of data for meta-analysis, more than half of the literature mentioned this tendency. We found the source of heterogeneity through sensitivity analysis, and there was no significant effect on the results before and after the removal of this literature, indicating that our results were robust. No obvious publication bias was found through Egger method (*P* = .672).

**Conclusion:**

People with ED have a higher risk of osteoporosis, especially among younger males. Because the assessment of osteoporosis is economical and noninvasive, ED patients should be evaluated by bone mineral density or men with osteoporosis should be further assessed for erectile function.

## 1 Introduction

Penile erection is the result of a complex neurovascular process involving the synchronic effects of vascular endothelium, smooth muscle, psychological, and neuroendocrine systems.^[1,2]^ Thus, good state and coordination of these events is essential for maintaining good erectile function, and any changes and disturbances may lead to erectile dysfunction (ED).^[3]^ ED is a very common male health problem, defined as the inability to achieve and maintain sufficient erections to achieve satisfactory sexual intercourse, affecting nearly a third of men over the age of 50.^[4]^ ED can have harmful effects on mental health, interpersonal relationships, and a wide range of psychosocial domains.^[5]^ A variety of ED-related risk factors have been identified, such as cigarette smoking, diabetes mellitus, cardiovascular events, chronic kidney disease, metabolic syndrome, and depression.^[6–11]^ Interestingly, osteoporosis also has similar risk factors, and several studies have suggested a correlation between ED and osteoporosis.^[12,13]^

Osteoporosis is a systemic metabolic bone disease characterized by impaired bone strength due to attenuated bone mineral density (BMD) and compromised bone quality, making patients prone to brittle fractures.^[14]^ Osteoporosis has previously been associated with postmenopausal women, and male osteoporosis patients have rarely been the focus of previous research.^[15,16]^ In fact, osteoporosis is also very common in men and is closely related to ED.^[17]^ Osteoporosis and ED significantly affect the quality of life in men.

As early as 2005, Keles et al^[17]^ proposed that there seemed to be a potential link between ED and osteoporosis. They found that the incidence of ED and osteoporosis increased with age, but the 2 appeared to be independent, with no increased risk associated with each other. Contrary to the results of Keles et al,^[17]^ another study on the relationship between ED and osteoporosis in 95 men with ED and 82 men with normal sexual function found that patients with ED had lower bone mineral density and a higher risk of osteoporosis than non-ED participants.^[18]^ A subsequent nationwide population-based retrospective cohort study also observed an increased risk of osteoporosis in ED patients, particularly among younger males (40–59 years).^[13]^ Although several articles have been published, whether ED increases the risk of osteoporosis, whether it can be an early predictor of osteoporosis, and the mechanisms underlying these relationships are still controversial. In this study, meta-analysis was used to further integrate and clarify the relationship between ED and osteoporosis, and the potential mechanism was integrated and systematically elaborated in order to provide ideas for future studies and guide clinical practice.

## 2 Methods

### 2.1 Search strategy

Two researchers independently conducted systematic retrieval of PubMed, EMBASE, Cochrane, and Web of Science, and the retrieval time was up to June4, 2020. The search terms used include (erectile dysfunction OR sexual dysfunction OR impotence) and (osteoporosis OR osteopenia OR bone mineral density OR bone density). We also browsed references of key articles and manually searched the gray literature to make sure no relevant articles were omitted. This system review and meta-analysis is reported in accordance with the preferred reporting items of the system review and meta-analysis (PRISMA).^[19]^ In addition, this study is a meta-analysis and does not involve ethical issues.

### 2.2 Inclusion and exclusion criteria

Inclusion criteria were as follows: The subjects were men with ED or osteoporosis. The study type was observational study. The outcome was the incidence of osteoporosis. There are clear, widely accepted diagnostic criteria for both ED and osteoporosis. The literature provided sufficient data to satisfy the completion of the meta-analysis.

Exclusion criteria were as follows: Outcomes were unclear and data were missing. Diagnostic criteria for osteoporosis and ED were unclear. Republished literature. Poor quality (Quality score less than 4^[20]^).

### 2.3 Selection process and data abstraction

The 2 reviewers first scanned the titles and abstracts independently for preliminary screening of all relevant literature. Literatures that initially meet the inclusion and exclusion criteria or that are controversial will be directly included in the full-text evaluation to ensure that all relevant papers are not omitted. At the full-text evaluation stage, disputes are negotiated by 2 reviewers, and if agreement cannot be reached, a third reviewer is consulted.

Two reviewers independently extracted baseline data and data required for meta-analysis using the pre-designed data extraction table. Baseline data extracted included first author and publication time, country, study type, age, body mass index (BMI), prevalence of diabetes, cases, ED-measurement, BMD-measurement, and quality score.

### 2.4 Methodological quality assessment

For included observational studies, including case--control studies, cohort studies, and cross-sectional studies, we used an 11-item checklist, which was recommended by Agency for Healthcare Research and Quality (AHRQ). If an item is answered “no” or “unclear,” it will be rated “0”; If the answer is “yes,” the score is “1.” Methodological quality of the literature can be assessed according to the total score: low quality = 0 to 3; moderate quality = 4 to 7; high quality = 8 to 11.

### 2.5 Statistical analysis

All statistical analyses and meta-analyses were performed using Cochrane Review Manager 5.3 (China) and Stata14 software, and the significance level was set at *P* < .05. We estimated the effect size of continuous variables by the mean difference (MD) and its 95% confidence interval (CI) and estimated the effect size of binary variables by the odds ratio (OR) of the calculated results and its 95% CI. Heterogeneity was evaluated using inconsistencies (*I*^2^) statistics. If *I*^2^ is greater than 50%, the heterogeneity is very significant and the random effect model should be adopted. If *I*^2^ is less than 50%, it indicates that the heterogeneity is within an acceptable range, and a fixed-effect model should be adopted.

### 2.6 Sensitivity analysis and publication bias

In order to find the source of heterogeneity, sensitivity analysis was conducted by eliminating each literature article one by one, recalculating and recording the heterogeneity and pooled effect values after removing a single study. After successively removing each study, we calculated the change of *I*^2^ through Revman5.3 and obtained the forest plot of sensitivity analysis through Stata14. After the source of heterogeneity is found, the target literature is analyzed in detail in order to find out the reason why it is the source of heterogeneity in this study.

Publication bias was assessed quantitatively by the Egger method. When the *P* value obtained by egger method is greater than .05, it means there is no significant publication bias. If *P* < .05, it indicated the existence of publication bias. In this case, the rim and fill method will be used to evaluate the impact of publication bias on our meta-analysis results. If publication bias is found to have a significant effect on results, we will discuss it in detail in our discussion.

## 3 Results

### 3.1 Literature retrieval results and basic characteristics

**Figure 1:**
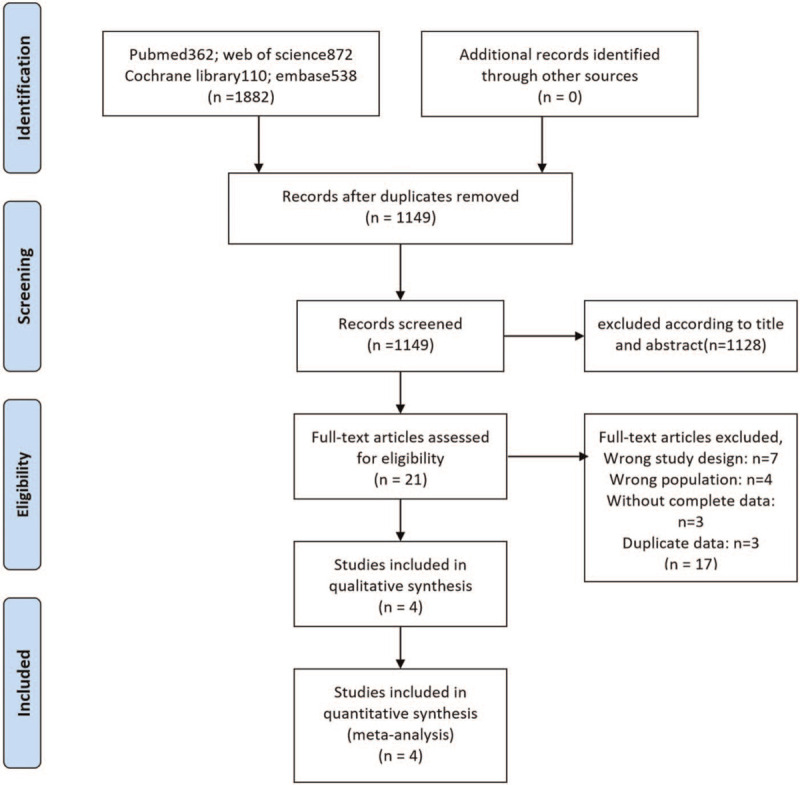
Literature search and selection process.

We searched a large number of literatures, carefully studied and screened them, and the specific process is shown in Fig. 1. According to the established retrieval formula, we searched a total of 1882 related studies, deleted duplicates and made preliminary screening according to titles and abstracts, and the remaining 21 literatures entered the full-text reading stage. After reading through the full text of 21 articles, a total of 4 studies^[12,13,17,18]^ including 22,672 participants were finally included in our meta-analysis. Among the 4 studies, 3 are cross-sectional^[12,17,18]^ and 1 is cohort.^[13]^ The baseline data of the studies included in the meta-analysis are summarized in Table 1.

**Table 1:** Characteristics of the included studies.

			Age					
Study	Country	Study type	ED group	Non-ED group	Cases (ED vs non-ED)	ED-measurement	BMD-measurement	Quality score
Nahas2017^[12]^	Malaysia	CS	50 (11.7)	46.9 (8.4)	90/29	IIEF-5	QUS	9/11
Wu2016^[13]^	China	Cohort	57.7 (10.2)	57.6 (10.7)	4460/17840	ICD-9-CM	ICD-9-CM	7/9
Dursun2015^[18]^	Turkey	CS	53.5 (38–69)	50.1 (31–69)	57/19	IIEF-5	DEXA	8/11
Keles2005^[17]^	Turkey	CS	59.9 ± 0.8	57.2 ± 0.2	95/82	IIEF	DEXA	7/11

### 3.2 Methodological quality assessment

We conducted a methodological quality assessment of the included studies using 11-item checklist recommended by Agency for Healthcare Research and Quality (AHRQ) and found that 2 articles were of high quality and the remaining 2 were of medium to high quality. Specific 11-item checklist and scoring values of various items can be found in Table 2.

**Table 2:** AHRQ checklist assessment of included studies.

	AHRQ 11-item checklist											
Study	Define the source of information	List inclusion and exclusion criteria	Indicate time period used for identifying patients	Indicate whether or not subjects were consecutive	whether evaluator covered up other aspects of the subject	Assessments for quality assurance purposes	Explain any patient exclusions from analysis	Describe how confounding was assessed	Explain how missing data were handled	Summarize patient response rates	Clarify percentage of incomplete follow-up data	Total
Nahas2017^[12]^	Y	Y	Y	Y	Y	Y	Y	N	N	Y	Y	10
Wu2016^[13]^	Y	Y	Y	N	Y	Y	N	Y	N	N	Y	9
Dursun2015^[18]^	Y	Y	N	N	Y	Y	Y	Y	N	Y	U	7
Keles2005^[17]^	Y	Y	Y	Y	Y	Y	Y	U	Y	Y	U	9

### 3.3 Meta-analysis results

**Figure 2:**
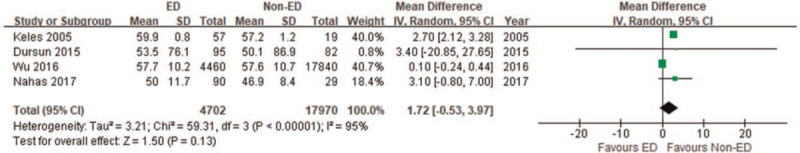
Forest plot-comparison of age between ED group and non-ED group.

**Figure 3:**
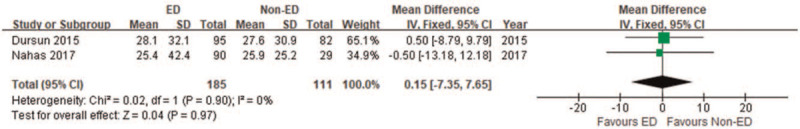
Forest plot-comparison of BMI between ED group and non-ED group.

**Figure 4:**
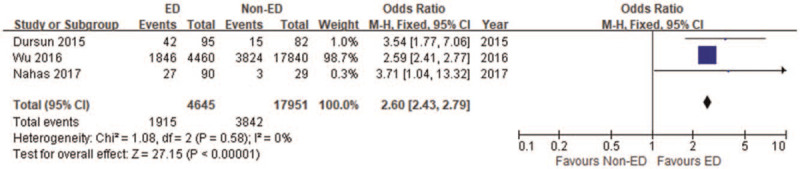
Forest plot-comparison of prevalence of diabetes between ED group and non-ED group.

We first performed a meta-analysis of baseline data that might be confounding factors affecting the stability of the main outcome, such as age and BMI. Four studies^[12,13,17,18]^ reported age data and two^[12,18]^ reported BMI data. Meta-analysis results showed that there was no significant difference in age (MD = 1.72, 95% CI -0.53 to 3.97, *P* = .13) (Fig. 2) and BMI (MD = 0.15, 95% CI -7.35 to 7.65, *P* = .97) (Fig. 3) between the ED group and the non-ED group. Three studies^[12,13,18]^ also reported the prevalence of diabetes, and our meta-analysis showed that the prevalence of diabetes in the ED group was significantly higher than that in the non-ED group (OR = 2.60, 95% CI 2.43–2.97, *P* < .001) (Fig. 4).

**Figure 5:**
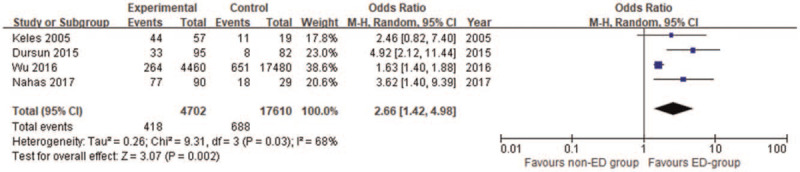
Forest plot-comparison of osteoporosis risk between ED group and non-ED group.

From the 4 studies,^[12,13,17,18]^ the relevant data of osteoporosis in the ED group and the non-ED group were extracted. We combined the included data through meta-analysis and found significant heterogeneity among groups (*I*^2^ = 68%). The meta-analysis results of the random-effect model showed that the incidence of osteoporosis in ED patients was significantly higher than that in the non-ED group (OR = 2.66, 95% CI 1.42–4.98, *P* = .002, *I*^2^ = 68%) (Fig. 5).

### 3.4 Sensitivity analysis

**Figure 6:**
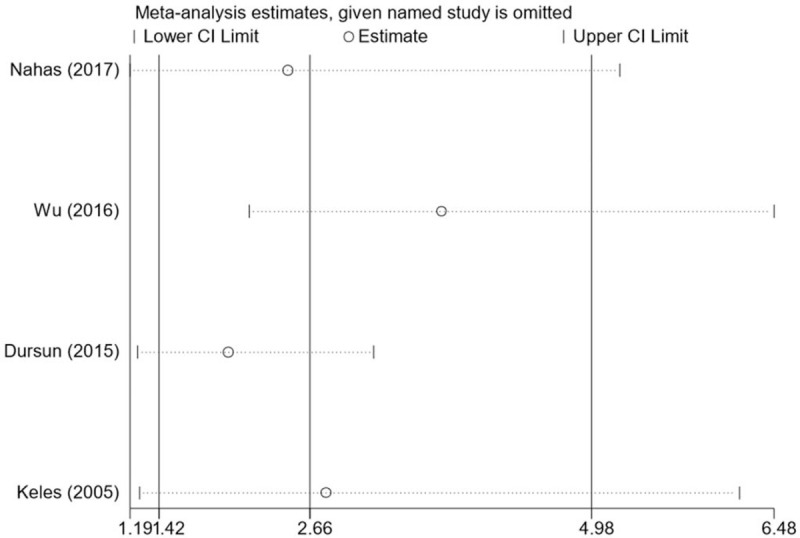
Forest plot for sensitivity analysis.

In our meta-analysis, the heterogeneity test results showed *I*^2^ = 68%, indicating significant heterogeneity. In order to find the source of heterogeneity, we removed each study in turn to conduct sensitivity analysis. When we removed Wu 2016,^[13]^ we found that *I*^2^ changed to 0, indicating that Wu 2016^[13]^ was likely to be the source of heterogeneity of this study (Table 3). Sensitivity analysis was also performed using Stata software and forest plots were drawn after each study was removed in order to visually discover the source of heterogeneity. As shown in Fig. 6, after the removal of Wu 2016, the pooled OR value was farthest from the median line, indicating that Wu 2016^[13]^ was likely to be the source of heterogeneity. However, as far as the OR value changes before and after the removal of Wu 2016^[13]^ were concerned, despite the existence of heterogeneity, the conclusions of the meta-analysis were not significantly affected.

**Table 3:** Sensitivity analysis.

Study omitted	*I*^2^	*P*	Pooled OR
Nahas2017^[12]^	71%	.02	2.49 [1.19–5.21]
Wu2016^[13]^	0%	<.0001	3.75 [2.17–6.48]
Dursun2015^[18]^	36%	.004	2.00 [1.25–3.19]
Keles2005^[17]^	78%	.01	2.80 [1.26–6.19]

### 3.5 Publication bias

**Figure 7:**
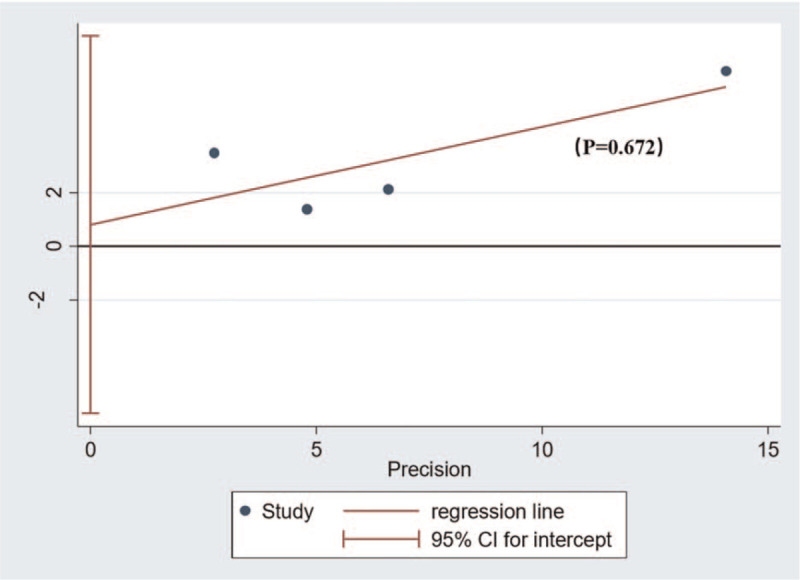
Publication bias - Egger graph.

We used egger method to quantitatively evaluate the publication bias of this study, and the results showed that *P* = .672, which was greater than .05, indicating that there was no obvious publication bias in this study (Fig. 7).

## 4 Discussion

As far as we know, our study is the first meta-analysis to explore the relationship between ED and osteoporosis, and the first systematic review to systematically elaborate the potential mechanism of the relationship between ED and osteoporosis. Meta-analysis results showed that compared with non-ED group, ED patients seemed to have a 2.66-fold higher risk of osteoporosis. Although our study was unable to combine the OR values after controlling for confounding factors due to insufficient data, in the study of Wu et al,^[13]^ after controlling for potential confounding factors, the risk of osteoporosis in the ED group was still 3.04 times that in the non-ED group, which was consistent with our results. Our meta-analysis of the baseline data also showed no significant differences in age and BMI between the groups, which to some extent excluded some possible confounding factors. In addition, both Wu et al^[13]^ and Nahas et al^[12]^ found in their study that compared with older participants, the increased risk of osteoporosis in ED patients seemed to be more obvious in younger participants. In general, our meta-analysis found that ED patients, especially those in the 40 to 59 age group, showed a high risk of osteoporosis.

The underlying mechanism of the relationship between ED and osteoporosis may be very complex, and several possible mechanisms have been proposed. First of all, ED patients are often accompanied by hypogonadism, and their natural free testosterone is often lower than that of non-ED men.^[21]^ Androgens can not only directly stimulate bone formation, but also reduce the accumulation of reactive oxygen species (ROS) in the body and slow down the apoptosis of osteoblasts and mesenchymal cells by combating oxidative stress, thus playing an important role in male bone formation.^[22,23]^ Multiple studies have shown a significant decrease in bone density and an increased risk of brittle fractures in patients with low testosterone levels.^[24,25]^ In addition, patients with prostate cancer who undergo androgen deprivation therapy or orchiectomy also experience decreased bone density and increased risk of fracture.^[26,27]^ There is also a link between the testosterone and vitamin D pathway.^[28]^ Testosterone deficiency is associated with decreased renal 1α-hydroxylase activity, which indirectly affects the parathyroid hormone-vitamin D axis, resulting in decreased 1,25-hydroxy vitamin D concentration.^[29]^ Vitamin D plays an important role in influencing bone metabolism and maintaining bone health, and several studies have shown that vitamin D deficiency leads to decreased bone density and an increased risk of osteoporosis and bone fractures.^[30,31]^ Thus, low testosterone levels and vitamin D deficiency in ED patients may be important mechanisms that increase the risk of osteoporosis.

ED is closely related to inflammation, and chronic low-level inflammation is an important component of ED, which may be a mediator of endothelial dysfunction.^[32]^ Studies have also shown that 5-item International Index of Erectile Function (IIEF-5) scores are negatively correlated with levels of several inflammatory markers. Inflammatory cytokines such as interleukin-1 (IL-1), interleukin-6 (IL-1), and tumor necrosis factor-alpha can damage the endothelial cells in the peripheral vascular bed of the penis, leading to vascular endothelial dysfunction and thereby promoting ED.^[32]^ These inflammatory cytokines may also be involved in regulating the function of osteoblasts and osteoclasts, thereby inhibiting bone growth and leading to osteoporosis.^[33,34]^ Nitric oxide (NO) bioactivity is essential for penile erection and is the main pathogenic mechanism of ED.^[35]^ Meanwhile, the decrease of NO may also lead to bone loss.^[36]^ Studies have found that NO has a biphasic effect on bone resorption. Low concentration of NO has been shown to enhance IL-1-induced bone resorption, while high level of NO has an inhibitory effect on bone resorption.^[37]^ Therefore, inflammatory bone loss in ED patients and the promotion of IL-1-induced bone resorption by low NO level may be the underlying mechanism of the relationship between ED and osteoporosis.

Depression may also be a key factor in explaining the relationship between ED and osteoporosis. There is a close interaction between depression and ED.^[38]^ Several studies have shown that depressed people have a higher risk of ED, and that people with ED are also more likely to have anxiety and even depression.^[39,40]^ Depression was also found to be a risk factor for osteoporosis.^[41]^ In depressed patients, hypothalamic–pituitary–adrenal axis is activated, and excessive secretion of corticotropin-releasing hormone leads to elevated cortisol levels. Cortisol can reduce the proliferation and differentiation of osteoblasts, promote the apoptosis of osteoblasts and osteoblasts, increase the excretion of calcium in urine, and reduce the absorption of calcium in the small intestine, all of which will lead to a decrease in bone density and thereby increase the risk of osteoporosis.^[42]^ Different from cortisol, growth hormone can act on osteoblasts and osteoclasts respectively to promote bone formation and absorption to play a role in bone remodeling and finally achieve the effect of bone accumulation. However, in depressed patients, the secretion of growth hormone is often reduced, and the somatotrophic axis is interrupted, thus affecting bone accumulation.^[42]^ It can be seen that ED patients are often accompanied by depression, which is also involved in the occurrence of osteoporosis.

A study based on the Japanese population pointed out that compared with older ED patients (55 years or more), younger ED patients (45–54 years old) had lower relationship satisfaction, lower job satisfaction, more negative reactions from sexual partners, more difficulties in adjusting to life with ED, and thus tended to have higher incidence of depression and more severe depressive symptoms.^[39]^ Interestingly, our study also found that the increased risk of osteoporosis in ED patients seemed to be more pronounced in younger participants (40–59 years old) than in older participants (60 years or more). Although the exact mechanism still needs to be studied, the consistency also suggests that depression plays an important role in the relationship between ED and osteoporosis.

Our sensitivity analysis found that Wu2016^[13]^ was the source of heterogeneity in this study. We compared Wu2016^[13]^ with other studies and found that its data came from the National Health Insurance Research Database (NHIRD), and the diagnosis of ED and osteoporosis was based on ICD-9-CM codes. When diagnosis is based on ICD-9-cm codes, the accuracy is often dependent on the performance of clinicians at the time. In addition, data such as body mass index, exercise ability, and dietary habits are often lacking in the database, which may have an impact on the study results. The above may be the reason why Wu2016^[13]^ became the source of heterogeneity in this study.

It is undeniable that our study has several limitations. First of all, only 4 studies were included, all of which were observational studies. Second, due to limited data, we were unable to conduct subgroup analysis of organic ED and psychogenic ED. Third, although we found that the increased risk of osteoporosis in ED patients seemed to be more pronounced in younger participants, we were unable to conduct a subgroup analysis by age due to data limitations. Fourth, although we have provided a comprehensive description of the possible underlying mechanisms, more clinical trials are needed to confirm the underlying mechanisms of this relationship.

## 5 Conclusion

People with ED have a higher risk of osteoporosis, especially among younger males. Doctors should be aware of this relationship in order to make appropriate recommendations to patients. Because the assessment of osteoporosis is economical and noninvasive, ED patients should be evaluated by bone mineral density or men with osteoporosis should be further assessed for erectile function. In order to further validate the relationship between ED and osteoporosis and to further explore its underlying mechanism, more prospective studies with large samples are needed for further study.

## Author contributions

**Acquisition, analysis, or interpretation of data:** All authors.

**Concept and design:** Jianglei Zhang, Jiangnan Xu, Chao Wang

**Conceptualization:** Jiangnan Xu, Chao Wang, Jianglei Zhang.

**Critical revision of the manuscript:** Jun Ouyang, Jianglei Zhang.

**Data curation:** Jiangnan Xu, Chao Wang, Yuhui Zhang, Zekun Xu, Jun Ouyang.

**Drafting of the manuscript:** Jiangnan Xu, Chao Wang.

**Formal analysis:** Jiangnan Xu.

**Software:** Jiangnan Xu.

**Statistical analysis:** Jiangnan Xu, Chao Wang, Yuhui Zhang.

**Writing – original draft:** Jiangnan Xu.

**Writing – review & editing:** Chao Wang, Yuhui Zhang, Zekun Xu, Jun Ouyang, Jianglei Zhang.
